# Cardiac myosin inhibitor, CK-586, minimally reduces systolic function and ameliorates obstruction in feline hypertrophic cardiomyopathy

**DOI:** 10.1038/s41598-024-62840-3

**Published:** 2024-05-27

**Authors:** Victor N. Rivas, Amanda E. Crofton, Carina E. Jauregui, Jalena R. Wouters, Betty S. Yang, Luke A. Wittenburg, Joanna L. Kaplan, Darren T. Hwee, Anne N. Murphy, Bradley P. Morgan, Fady I. Malik, Samantha P. Harris, Joshua A. Stern

**Affiliations:** 1 Department of Clinical Sciences, College of Veterinary Medicine, North Carolina State University, 1060 William Moore Dr, Raleigh, NC 27607 USA; 2 Department of Medicine and Epidemiology, School of Veterinary Medicine, University of California-Davis, Davis, CA USA; 3 Department of Surgical and Radiological Sciences, School of Veterinary Medicine, University of California-Davis, Davis, CA USA; 4 Research and Non-Clinical Development, Cytokinetics, Inc., South San Francisco, CA USA; 5 Department of Physiology, University of Arizona, Tucson, AZ USA

**Keywords:** Pharmacodynamics, Left ventricular outflow tract obstruction (LVOTO), Obstructive hypertrophic cardiomyopathy (oHCM), Cat, Myosin-inhibitor, Cardiology, Cardiomyopathies, Preclinical research, Translational research

## Abstract

Hypertrophic cardiomyopathy (HCM) remains the most common cardiomyopathy in humans and cats with few preclinical pharmacologic interventional studies. Small-molecule sarcomere inhibitors are promising novel therapeutics for the management of obstructive HCM (oHCM) patients and have shown efficacy in left ventricular outflow tract obstruction (LVOTO) relief. The objective of this study was to explore the 6-, 24-, and 48-hour (h) pharmacodynamic effects of the cardiac myosin inhibitor, CK-586, in six purpose-bred cats with naturally occurring oHCM. A blinded, randomized, five-treatment group, crossover preclinical trial was conducted to assess the pharmacodynamic effects of CK-586 in this oHCM model. Dose assessments and select echocardiographic variables were assessed five times over a 48-h period. Treatment with oral CK-586 safely ameliorated LVOTO in oHCM cats. CK-586 treatment dose-dependently eliminated obstruction (reduced LVOTOmaxPG), increased measures of systolic chamber size (LVIDs Sx), and decreased select measures of heart function (LV FS% and LV EF%) in the absence of impact on heart rate. At all tested doses, a single oral CK-586 dose resulted in improved or resolved LVOTO with well-tolerated, dose-dependent, reductions in LV systolic function. The results from this study pave the way for the potential use of CK-586 in both the veterinary and human clinical setting.

## Introduction

Hypertrophic cardiomyopathy (HCM) remains the most common heritable cardiomyopathy of humans affecting approximately 1 in every 500 people^1^. The disease is largely considered genetic, most commonly caused by mutations in genes encoding sarcomeric proteins with a resultant pathogenic effect of sarcomeric hypercontractility^2^. This aberrant dynamic change predominantly affects the left ventricular (LV) chamber of the heart and leads to concentric hypertrophy, myocardial fibrosis, and myofiber disarray^3^. In the case of severe LV hypertrophy or asymmetric septal hypertrophy (ASH), hyperdynamic LV contractility causes systolic anterior motion of the mitral valve resulting in obstruction to LV outflow and LV pressure overload (obstructive HCM [oHCM])^4^. Obstruction at rest (without provocation) is observed in ~ 20–33% of human HCM patients and is associated with increased disease morbidity (e.g., exercise intolerance, syncope, angina, and fatigue) and severe disease sequalae (i.e., left-side congestive heart failure [CHF], thromboembolic disease, and sudden cardiac death) inherently decreasing patient quality-of-life^5–10^.

Hypertrophic cardiomyopathy is seen at an even greater prevalence in cats, affecting up to 14% of the general cat population^11–14^. Like humans, the etiology of the disease has a genetic basis; however, all previously reported feline HCM-causing mutations remain breed-specific (i.e., Maine Coons, Ragdolls, and Sphynx) and explain only a small portion of total feline HCM cases^15–17^. Left ventricular outflow tract obstruction (LVOTO) is observed in ~ 33–63% of HCM-affected cats; yet, unlike oHCM-affected human patients, the presence of LVOTO has not yet been linked to increased disease morbidity in cats^13,14,18,19^. Despite the aforementioned clinical differences in disease manifestations, cats closely recapitulate the genetic, pathophysiologic, and hemodynamic aspects of human HCM. As such, genetic feline models of HCM and oHCM provide a unique opportunity for the study of novel therapeutics aimed to treat features of HCM, including LVOTO^20^.

Small-molecule inhibitors that modulate the sarcomere are promising novel therapeutics for LVOTO management in oHCM patients. However, the availability of such pharmaceuticals is limited in both human and veterinary patients. In addition to the use of surgical or interventional procedures (e.g., surgery and alcohol septal ablation), where incomplete response and risk of in-hospital morbidity and mortality is relatively high^21–23^, mavacamten is currently the only FDA-approved cardiac sarcomere inhibitor treatment option for symptomatic oHCM. The drug’s mechanism of action is to bind to the cardiac sarcomere myosin heads and reduce sarcomeric ATPase activity, consequently decreasing the myocardial hypercontractility that leads to outflow tract pressure gradients and LVOTO^24^. The novel compound, aficamten, is in Phase-III human clinical trial investigation at the time of this writing. Neither mavacamten nor aficamten are FDA-approved for use in animals. In feline HCM or oHCM, pharmaceutical management of LVOTO is limited to the use of beta-blockers (e.g., atenolol) which have not been shown to improve feline quality-of-life or lessen mortality^25,26^. Collectively, this represents a dire need for novel pharmaceuticals for the treatment of HCM and oHCM in human and veterinary patients alike.

We previously demonstrated successful acute dose-dependent (0.30- and 1.0 mg/kg) reductions in LV systolic function, left ventricular outflow tract maximum pressure gradient (LVOTmaxPG), and isovolumetric relaxation times (IVRT) after of a single oral gavage dose of aficamten in cats affected by asymptomatic oHCM harboring at least one copy of the feline A31P *myosin-binding protein C3* (*MYBPC3*) mutation^27,28^. Aficamten was well tolerated by all cats receiving treatment and successfully improved LVOTO. In this study, we embarked on the first-ever examination of the 6-, 24-, and 48-hour (h) pharmacodynamic effects of an oral formulation of a novel cardiac myosin inhibitor, CK-4021586 (CK-586), in six purpose-bred cats with naturally occurring feline oHCM. We hypothesized that oral administration of CK-586 in cats afflicted by oHCM will result in dose-dependent relief of LVOTO, and that the drug will be safe and well tolerated. The results of this study highlight the promising pharmacodynamic effects of CK-586 for alleviation of obstruction in feline HCM.

## Material and methods

### Ethics statement

All procedures were approved by the Institutional Animal Care and Use Committee (IACUC) of the University of California-Davis (Davis, CA, USA) and carried out in accordance with guidelines and regulations (protocol #22376). Additionally, this study was executed in compliance with the ARRIVE 2.0 guidelines, the Animal Welfare Act, and the Institute for Laboratory Animal Research Guide for the Care and Use of Laboratory Animals^29–31^.

### Pharmacokinetic study dose determinations and sample analyses

**Figure 1:**
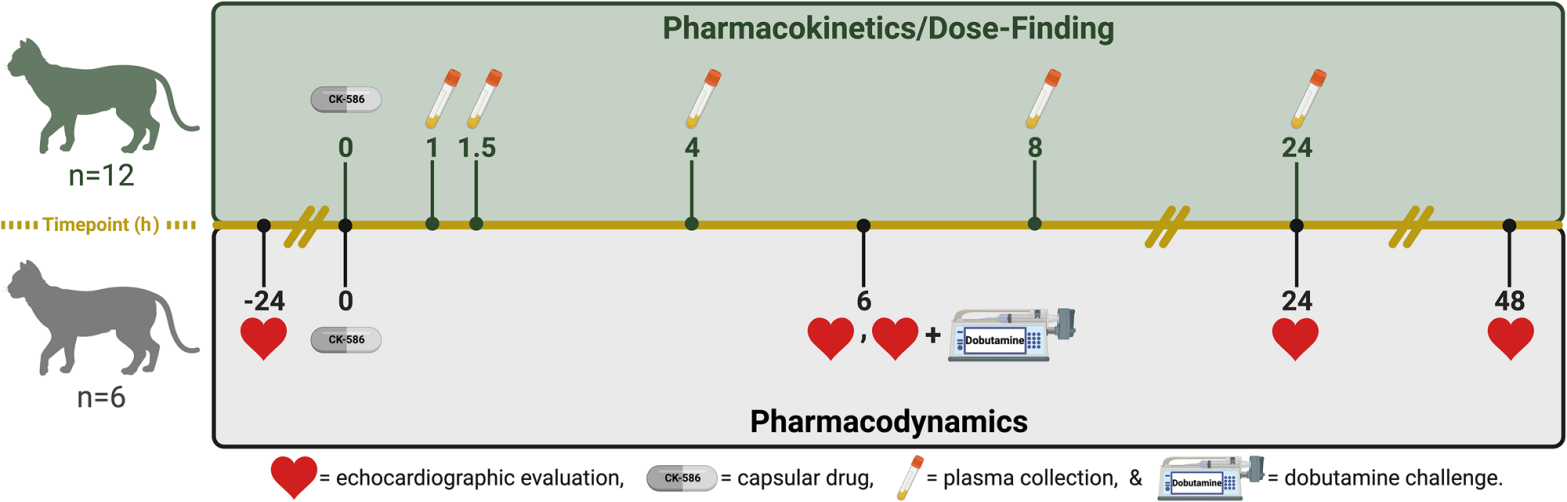
Study design and timepoints. Visual representation of study design and timepoints for the pharmacokinetic/dose-finding (top green) and pharmacodynamic (bottom gray) phases of the study is presented. *h* hour.

Eight months prior to the echocardiography study, 12 apparently healthy cats, free of any cardiac and metabolic disease confirmed by board certified veterinary cardiologists (J.L.K. and J.A.S.), were selected to assess the pharmacokinetic profile of CK-586 and determine doses for subsequent pharmacodynamic investigations of CK-586. Eight cats in this cohort were equally randomized and dosed with either a low- (3 mg/kg) or high-dose (10 mg/kg) of capsular CK-586 as a single oral dose. Four additional dose levels (4.8-, 10.5-, 15.9-, and 16.2 mg/kg) of CK-586 were given to four additional cats to assess the drug profiles at altered concentrations. All cats were sedated with a combination of 2- and 0.30 mg/kg of alfaxalone and midazolam, respectively, for placement of a jugular sampling catheter. Cats were monitored and allowed to fully recover from conscious sedation (a period of approximately 1.5 h); subsequently, a single randomized oral dose of CK-586 was administered. Cat blood was obtained at 1-, 1.5-, 4-, 8-, and 24 h after dosing from the jugular sampling catheter via standard methodology into 2 mL EDTA tubes (Fig. 1). Whole-blood EDTA samples were processed to plasma via centrifugation (1000 × g for 15 min) and stored in − 80 °C for subsequent drug profiling.

Total CK-586 plasma concentrations were measured in a single batch by routine liquid chromatography-tandem mass spectrometry (LC-MS/MS) methodology as previously described^28^. Briefly, a 40 µL aliquot of each plasma was mixed with 120 µL of acetonitrile that contained N1-(butylcarbamoyl)-sulfanamide (0.1 µM) as the internal standard (retention time = 1.29 min). The mixture was then vortexed and centrifuged. The resulting supernatant was transferred and filtered through a membrane (Pall Corporation AcroprepAdv 96-well Filter Plate, 0.2 µm WWPTFE membrane), then diluted 1:1 with HPLC grade water. Ten µL of the resulting solution was injected onto a reverse-phase C18 column, and the resultant peaks detected on a SCIEX API 4000 LC-MS/MS equipped with a turbo ion spray ionization source. Mobile Phase A was 0.1% formic acid in water and Phase B was 0.1% formic acid in acetonitrile. The lower limit of quantification and retention time for CK-586 was 1 ng/mL and 1.48 min, respectively.

### Pharmacodynamics study animals and design

The pharmacodynamic effects of CK-586 delivered as an active pharmaceutical ingredient (API) in capsule form was assessed as a blinded, randomized, five-treatment group, crossover preclinical trial in a purpose-bred feline model of oHCM^20^. A total of six ACVIM Stage B1 HCM-confirmed cats (i.e., subclinical LV hypertrophy without left atrial [LA] enlargement) with concomitant LVOTO, diagnosed by a board-certified veterinary cardiologist (J.A.S.), were initially randomized into one of three treatment groups (Vehicle, 5-, and 10 mg/kg). Following interim data analysis, cats were later again randomized to a lower (2 mg/kg) and higher (15 mg/kg) dose group. Cat Stage B1 HCM-affection status was confirmed by an echocardiographic diagnosis following ACVIM consensus guideline inclusion criteria (i.e., 2D or M-mode diastolic diameter of the interventricular septum or LV posterior wall > 6 mm, excluding insertion sites of moderator bands, with normal LA size confirmed by a left atrial-to-aortic root ratio [LA:Ao] measurement < 1.5) with concomitant LVOTO as evidenced by a left ventricular outflow tract (LVOT) velocity > 1.9 m/s (LVOTmaxPG ≥ 14.5 mmHg)^12,32–34^. HCM-affected cats between one and 10 years-of-age, with no evidence of CHF, and deemed to be otherwise healthy (i.e., clear from metabolic disease and systemic hypertension as a component of routine cat colony protocol by ensuring a Doppler blood pressure < 160 mmHg, normal fundic examination, and normal serum biochemical profile confirming absence of renal disease), were included in the study. Baseline primary data and sample collections were obtained one day prior to CK-586 administration. Cats received a single dose of assigned treatment and additional data was collected 6-, 24-, and 48 h post-treatment (Fig. 1). In addition to standard timepoint evaluation, intravenous (IV) challenge with a 10 mcg/kg/min dose of dobutamine was performed for all cats at the 6 h timepoint to increase heart rate (HR) and contractility, provoking LVOTO (‘6 h Challenge’). All cats had an average washout period of 18.5 days between treatment groups (7-day minimum and 35-day maximum), and subsequently re-randomized until all six cats had successfully received treatments at all doses previously described. With the exception of the treatment dispenser (C.E.J.), all study participants were blinded for all aspects of the study. Demographic information for all cats used in this study are presented in Supplemental Table 1.

**Table 1:** Within-dose changes in measures of heart chamber size and function between baseline and study timepoints.

Timepoint	HR	RPLA LA (mm)	LVIDd Sx (mm)	LVIDs Sx (mm)	LV FS%	LV EF%	LVOTmaxPG (mmHg)	MV E/A	LV IVRT (msec)	Lau Flow (cm/sec)
Vehicle										
Baseline	169 (141–230)	13.85 (12.8–14.9)	12.8 (10.8–15.6)	5 (3.7–6)	56.95 (48.8–75.9)	90 (84.2–98)	3.175 (1.68–3.86)	1.135 (0.69–1.61)	52 (42–65)	43.09 (39.93–48.26)
6 h Post Drug	170.5 (153–210)	13.75 (12.5–15)	13.6 (8.4–15.4)	5.8 (4.5–9.1)	50.8 (41.2–61.5)	85.6 (75.8–92.7)	3.055 (2.31–13.02)	0.99 (0.67–1.58)	61.5 (49–83)	42.12 (33.78–48.26)
6 h Challenge	237 (174–260)*	14.1 (13.6–15.8)	13.2 (10.6–15.9)	2.8 (1.9–3.2)	80.65 (75.4–86)*	98.95 (98–99.6)*	33.41 (15.17–135.4)**	0.825 (0.69–1.57)	44 (38–46)	54.85 (38.17–68.01)
24 h Post Drug	171.5 (151–226)	13.1 (11.7–15.1)	13.6 (11.8–15)	5.5 (4.3–8.2)	58.5 (43.9–66.3)	90.85 (78.8–95)	5.425 (2.25–17.13)	1.31 (0.7–2.26)	56.5 (40–69)	40.15 (35.98–58.35)
48 h Post Drug	174.5 (135–205)	13.75 (12.6–15.5)	12.85 (10–16.1)	5.25 (4.3–7.6)	55.5 (53–64.6)	89.25 (86.9–94.2)	14.28 (4.14–64.89)	1.085 (0.93–2.07)	59.5 (49–76)	48.27 (41.68–72.65)
**Friedman *P*-value**	**0.0138**	0.1138	0.7847	**0.0086**	**0.0036**	**0.0041**	**0.0008**	0.548	**0.0028**	0.3624
2 mg/kg										
Baseline	164 (134–238)	13.35 (12.3–16.2)	13.25 (8.6–15.4)	6.25 (3.8–8.7)	52.85 (40–59.9)	87 (74.6–91.5)	3.69 (0.65–17.64)	0.975 (0.76–1.35)	65 (43–75)	50.02 (29.83–55.28)
6 h Post Drug	156 (122–218)	14.05 (11.9–14.3)	12.6 (10.8–15.8)	6.85 (3.1–10.4)	51.05 (34.5–71)	85.35 (67.5–96.9)	3.195 (1.57–8.08)	0.985 (0.7–1.76)	60 (50–85)	44.53 (32.47–46.51)
6 h Challenge	214.5 (180–245)	14.05 (11.7–16.5)	12.95 (12.2–16.8)	5.2 (2.6–7.4)	65.25 (39.1–79)	94.4 (74–98.7)	30.33 (9.88–57.88)*	0.905 (0.75–1.35)	46.5 (39–55)	56.16 (35.98–72.52)
24 h Post Drug	159 (136–226)	13.4 (12.5–15.9)	12.25 (11.2–15)	5.7 (5.2–9.1)	51.95 (39.5–63.3)	86.55 (73.9–93.5)	2.755 (1.67–8.96)	0.89 (0.8–1.17)	53 (48–71)	43.66 (34.22–50.02)
48 h Post Drug	163.5 (143–235)	13.55 (11.9–16.4)	12.3 (10.7–14.4)	5.75 (4.2–8.6)	54.2 (40.4–63.3)	88.2 (75–93.8)	12.59 (1.8–39.92)	0.855 (0.73–1.25)	55 (44–71)	49.36 (39.49–56.16)
**Friedman *P*-value**	0.0708	0.9787	0.1904	0.2416	0.2311	0.2942	**0.0289**	0.3297	**0.0336**	0.1346
5 mg/kg										
Baseline	166 (126–233)	13.15 (11.6–14.9)	12.7 (10.5–15.2)	5.9 (4.9–7.8)	51.65 (47–59.3)	86.3 (82.6–91.5)	1.905 (1.57–4.98)	1.05 (0.82–2.63)	56 (46–60)	48.04 (42.12–57.04)
6 h Post Drug	170.5 (154–210)	13.55 (11.3–15)	13.2 (10.1–16.6)	8.35 (4.7–10.6)	38.1 (32.2–53.8)	72.1 (64.5–88.2)	2.035 (0.79–4.22)	1.16 (0.77–2.83)	56 (48–70)	33.13 (30.27–50.02)
6 h Challenge	209 (183–245)	15.45 (12.2–17.8)**	12.65 (12–17.1)	5.8 (1.6–7.4)	58.9 (51.2–87.1)	90.85 (85.9–99.7)	16.3 (8.14–28)*	0.84 (0.73–1.1)	41.5 (32–63)	46.66 (42.56–59.23)
24 h Post Drug	163.5 (128–208)	13.75 (11.9–16)	13.2 (11–15)	7.45 (5.6–9.5)	38.05 (36.1–62.1)	72.65 (70–92.9)	1.905 (1.22–6.24)	1.175 (0.87–1.51)	60.5 (50–76)	42.12 (30.71–61.42)
48 h Post Drug	157.5 (128–218)	14.05 (13–15.1)	13.1 (10.5–15.3)	5.9 (3.3–9.8)	50.25 (35.5–73.5)	85.25 (68.9–97.5)	4.135 (2.11–24.85)	1.2 (0.74–2.07)	56 (47–72)	49.8 (30.71–68.01)
**Friedman *P*-value**	**0.0018**	**0.0184**	0.8595	0.0966	**0.0404**	**0.0323**	**0.0065**	0.434	**0.0168**	0.2796
10 mg/kg										
Baseline	166 (139–238)	12.8 (11.4–15.3)	12.45 (10.5–14.4)	6.4 (4.7–8.1)	50.1 (39–56.1)	84.7 (74.2–89.3)	3.63 (1.51–23.98)	1.075 (0.76–1.34)	59.5 (37–64)	47.17 (40.8–57.91)
6 h Post Drug	174 (150–218)	14.5 (12.9–16.3)*	13.25 (10.7–15.3)	8.3 (5.5–9.7)	38.4 (31.2–57.3)	72.7 (63.3–90.3)	1.715 (0.99–4.89)	1.085 (0.78–1.25)	54 (44–74)	45.85 (32.03–56.16)
6 h Challenge	227 (191–262)*	15.45 (13.8–18.5)***	13.4 (11.9–15.3)	6.5 (1.7–8.1)	51.9 (42.4–87.5)	86.1 (77.7–99.7)	9.355 (3.84–31.26)	0.98 (0.81–1.25)	43 (33–46)	48.92 (44.31–66.69)
24 h Post Drug	164.5 (114–226)	13.85 (13.7–15.6)	13.65 (11.1–14.9)	8.55 (5.4–9.6)	38.05 (35.4–53.3)	72.35 (68.9–87.6)	2.92 (1.32–9.01)	1.38 (0.82–2.04)	56.5 (45–73)	39.71 (35.98–53.09)
48 h Post Drug	172 (157–216)	14 (12.7–16.5)	13.25 (10.5–13.9)	6.1 (4.9–8.9)	48.45 (36.2–58.5)	83.7 (70.1–90.9)	3.13 (1.43–25.95)	1.265 (0.8–2.12)	54.5 (43–71)	41.9 (36.85–60.08)
**Friedman *P*-value**	0.0628	**0.0014**	0.1559	**0.0468**	0.0628	**0.0362**	**0.0116**	0.2934	**0.0307**	0.3496
15 mg/kg										
Baseline	189.5 (137–227)	14.55 (11.7–15.4)	12.5 (10.2–14.1)	6.05 (4.8–8.9)	47.8 (35–59.6)	83.1 (68.6–91.8)	4.385 (1.47–27.17)	0.895 (0.75–0.97)	52.5 (48–56)	48.48 (34.02–62.74)
6 h Post Drug	179 (159–223)	14.4 (13.7–15.4)	12.65 (11–15)	8.1 (7.6–10.6)*	30.4 (25–46.5)*	62.2 (53.8–81.5)*	1.885 (0.92–2.72)*	0.855 (0.79–1.63)	58.5 (48–79)	49.14 (36.85–57.04)
6 h Challenge	210.5 (199–268)	15.2 (14.1–16.4)	13.65 (10.2–16.3)	6.35 (4.4–9.8)	51.9 (37.7–60.7)	86.25 (72.7–92.5)	9.68 (6.69–23.67)	0.87 (0.78–0.98)	48.5 (38–53)	58.61 (41.24–78.3)
24 h Post Drug	183.5 (154–202)	14.3 (12.8–15.7)	13.15 (10.5–15)	7.75 (5.3–18.2)	41.35 (30.3–50)	76.35 (61.6–85.2)	2.105 (1.31–9.68)	0.935 (0.78–1.46)	62 (52–73)	42.56 (36.85–60.55)
48 h Post Drug	176.5 (148–188)	13.9 (12.8–15.8)	12.55 (10.7–14.1)	6 (3.8–8.4)	50 (40.2–64.7)	85.15 (74.8–94.5)	4.385 (1.43–29.52)	0.98 (0.7–1.31)	56.5 (50–74)	49.36 (41.24–54.84)
**Friedman *P*-value**	**0.0258**	**0.0404**	0.5636	**0.0142**	**0.0061**	**0.0087**	**0.0027**	0.7759	**0.0404**	0.5037

Results from a Friedman’s ANOVA analysis is presented. Friedman test *P*-values are reported for all comparisons within dose groups; bolded and asterisked *P*-values represent statistically significant Dunn’s pairwise comparisons for each timepoint compared to Baseline values. * = *P* < 0.05; ** = *P* < 0.01; *** = *P* < 0.001.

*HR* heart rate, *RPLA LA* maximal right parasternal long-axis left atrial diameter, *LVIDd Sx* short-axis diastolic left ventricular internal diameter, *LVIDs Sx* short-axis systolic left ventricular internal diameter, *LV FS%* percent left ventricular fractional shortening, *LV EF%* percent left ventricular ejection fraction, *LVOTmaxPG* left ventricular outflow tract maximum pressure gradient, *MV E/A* mitral valve passive filling/active filling ratio, *IVRT* isovolumetric relaxation time, *Lau* left auricular flow velocity, *h* hour.

### Echocardiographic evaluations

On the days of echocardiographic evaluation, a single 100 mg dose of oral gabapentin was given to all study participants an hour prior to complete echocardiographic assessment. All cats were sedated with a combination of 2- and 0.30 mg/kg of alfaxalone and midazolam, respectively, for all study echocardiographic examinations. Routine 2D, M-mode, Color and Spectral Doppler echocardiography was performed to acquire measurements of chamber size, LV wall thickness, and systolic function parameters from the right parasternal long-axis (RPLA) imaging window. Using the left-apical imaging window, indices of diastolic function and LVOTmaxPG were interrogated. Briefly, the following selected measures were recorded using previously described methodology and an off-cart analysis software (Syngo Dynamic Workplace, Siemens Medical Solutions, Malvern, PA, USA): HR, maximal right parasternal long-axis left atrial diameter (RPLA LA), short-axis diastolic left ventricular internal diameter (LVIDd Sx), short-axis systolic left ventricular internal diameter (LVIDs Sx), percent left ventricular fractional shortening (LV FS%), percent left ventricular ejection fraction (LV EF%), LVOTmaxPG, mitral valve passive filling/active filling ratio (MV E/A), isovolumetric relaxation time (IVRT), and left auricular flow velocity (Lau)^33^. Measurements were recorded as an average of three consecutive cardiac cycles when possible, avoiding any cycles during or immediately following cardiac arrhythmias. Cats were monitored for a period of at least three hours after each echocardiographic examination and returned to the colony following confirmation of full recovery from conscious sedation.

A concomitant lead-II electrocardiogram (ECG) was acquired throughout echocardiography to ensure measurements obtained avoided arrhythmic cycles and no arrythmias requiring therapy were present. All echocardiograms were performed and measured by a single, treatment and subject-blinded, board-certified veterinary cardiologist (J.A.S.).

### Statistical analyses

Pharmacokinetic parameters for CK-586 were examined following oral dosing and were estimated by noncompartmental analysis using the commercially available software program Phoenix® WinNonlin® (v8.0, Certara Inc., Princeton, NJ, USA). Pharmacokinetic parameters are reported as mean with standard deviation. Plasma CK-586 concentration–time graphs were generated using Prism Software (GraphPad, San Diego, CA, USA).

D’Agostino-Pearson, Anderson–Darling, and Kolmogorov–Smirnov normality testing was performed for all echocardiographic variables; all echocardiographic variables in this study were treated as non-normally distributed. To determine treatment-related changes within each dose group in HR, measures of chamber size (RPLA LA, LVIDd Sx, LVIDs Sx), and indices of heart function (LV FS%, LV EF%, LVOTmaxPG, MV E/A, LV IVRT, and Lau) at each dose (Vehicle, 2-, 5-, 10-, and 15 mg/kg), a Friedman’s analysis of variance (ANOVA) was employed with Dunn’s multiple comparisons testing, where the mean rank of each timepoint was compared to the mean rank of Baseline values. For each variable with a significant Friedman’s *P*-value, a two-way repeated measures ANOVA to fit a full model between dose groups (column effect: dose; row effect: timepoint; and column/row interactions were evaluated) with Geisser-Greenhouse correction and Dunnett’s multiple comparisons test was performed. This testing compared means of treatment (2-, 5-, 10-, and 15 mg/kg) with control means (Vehicle). A 2 × 2 contingency table was constructed and Fisher’s exact test was executed to assess differences between the observed incidence of LVOTO at every timepoint where cats were receiving treatment as opposed to the number of times obstruction was evidenced when cats were given Vehicle. Echocardiographic data was analyzed using Prism software (GraphPad, San Diego, CA, USA) where results were considered significant at a *P*-value < 0.05.

## Results

### Safety and tolerability

CK-586 (2–15 mg/kg) was safe and well tolerated in cats with oHCM. No adverse events along with no evidence of critically reduced LV EF% across all doses were recorded throughout all aspects of the study.

### Pharmacokinetics/dose-finding

**Figure 2:**
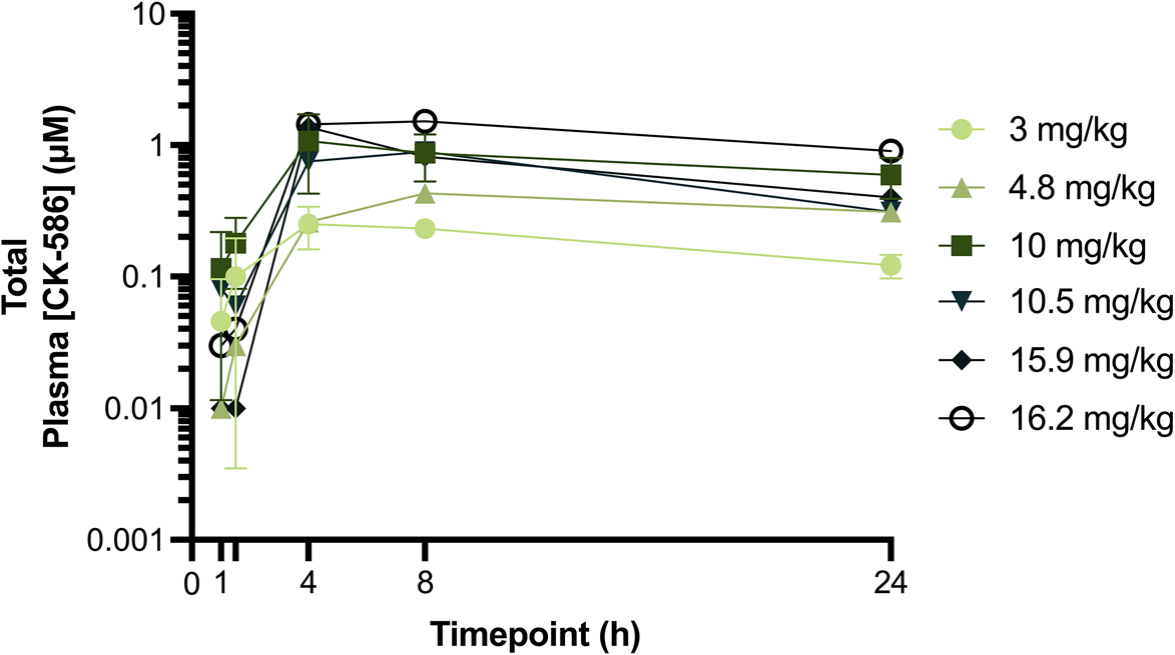
Plasma [CK-586] pharmacokinetic/dose-finding analysis. Mean total plasma [CK-586] values across four cats in the 3- and in the 10 mg/kg dose groups, as well as a single cat in the 4.8-, 10.5-, 15.9-, and 16.2 mg/kg dose groups spanning a 24 h period immediately following a single oral dose delivered as an API in capsule are illustrated. *h* hour, *API* active pharmaceutical ingredient.

At the timepoints evaluated, the mean maximum concentration (C_max_) for total plasma CK-586 was 1.21 µM and 0.28 µM in the 10- and 3 mg/kg dose groups, respectively (Fig. 2 and Supplemental Table 2). The average time-to-maximum plasma concentration (T_max_) across all cats is estimated to be 6.6 h (± 6.0) (Supplemental Table 2). Using this pharmacokinetic data from the 12 previously mentioned healthy cats and *ex-ante* pharmacodynamic data in healthy rats (Supplemental Fig. 1), it was determined that an initial 5- and 10 mg/kg dose of CK-586 would be used to assess the pharmacodynamic effects of the drug on LVOTO amelioration followed by interim analysis and subsequent dose selection of higher and lower dose groups.

**Table 2:** Between-timepoint changes in measures of heart chamber size and function between vehicle and treatment doses.

Comparisons	HR	RPLA LA	LVIDs Sx	LV FS%	LV EF%	LVOTmaxPG	LV IVRT
Baseline							
Vehicle vs. 2 mg/kg	–	–	–	–	–	–	–
Vehicle vs. 5 mg/kg	–	–	–	–	–	–	–
Vehicle vs. 10 mg/kg	–	–	–	–	–	–	–
Vehicle vs. 15 mg/kg	–	–	–	–	–	–	–
6 h Post Drug							
Vehicle vs. 2 mg/kg	–	–	–	–	–	–	–
Vehicle vs. 5 mg/kg	–	–	–	–	–	–	–
Vehicle vs. 10 mg/kg	–	–	–	–	–	–	–
Vehicle vs. 15 mg/kg	–	–	–	**0.0111**	**0.0106**	–	–
6 h Challenge							
Vehicle vs. 2 mg/kg	–	–	**0.0369**	–	–	–	–
Vehicle vs. 5 mg/kg	–	–	–	–	–	–	–
Vehicle vs. 10 mg/kg	–	–	–	–	–	–	–
Vehicle vs. 15 mg/kg	–	–	**0.005**	**0.0004**	**0.0154**	–	–
24 h Post Drug							
Vehicle vs. 2 mg/kg	–	–	–	–	–	–	–
Vehicle vs. 5 mg/kg	–	–	–	–	–	–	–
Vehicle vs. 10 mg/kg	–	–	–	**0.0282**	**0.0209**	–	–
Vehicle vs. 15 mg/kg	–	–	–	–	–	–	–
48 h Post Drug							
Vehicle vs. 2 mg/kg	–	–	–	–	–	–	–
Vehicle vs. 5 mg/kg	–	–	–	–	–	–	–
Vehicle vs. 10 mg/kg	–	–	–	–	–	–	–
Vehicle vs. 15 mg/kg	–	–	–	–	–	–	–
**ANOVA *P*-value**	0.9368	0.8430	0.0786	**0.0024**	**0.0034**	0.1307	0.9206

Results from a full model two-way repeated measures ANOVA test are presented. ANOVA *P*-values are reported for all comparisons across treatment groups; *P*-values for only the statistically significant Dunnett’s multiple comparisons test for each dose group compared to Vehicle values are reported. Bolded *P*-values represent statistically significant results at an alpha level of 0.05.

*HR* heart rate, *RPLA LA* maximal right parasternal long-axis left atrial diameter, *LVIDs Sx* short-axis systolic left ventricular internal diameter, *LV FS%* percent left ventricular fractional shortening, *LV EF%* percent left ventricular ejection fraction, *LVOTmaxPG* left ventricular outflow tract maximum pressure gradient, *IVRT* isovolumetric relaxation time, *Lau* left auricular, *h* hour.

### Heart rate

*Comparisons within treatment groups to baseline*: After dobutamine challenge, all dose groups showed a notable increase in HR with medians exceeding 200 beats per minute (bpm) for all groups. Heart rate values were significantly higher in the Vehicle and 10 mg/kg dose group after dobutamine challenge (*P*_adjusted_ = 0.0139 and *P*_adjusted_ = 0.0423, respectively; Table 1).

*Comparisons between treatment groups to vehicle*: Vehicle and all other dose group HR values were not different from each other at any timepoint. The overall ANOVA *P*-value for HR was 0.9368 (Table 2).

### Chamber size

#### LA diameter in right parasternal long-axis

*Comparisons within treatment groups to baseline*: In the 5- and 10 mg/kg treated cats, LA chamber size values significantly increased from Baseline at the 6 h Challenge timepoint (*P*_adjusted_ = 0.0076 and *P*_adjusted_ = 0.0002, respectively). 6 h Post Drug RPLA LA values were significantly higher compared to Baseline in the 10 mg/kg group (*P*_adjusted_ = 0.0247; Table 1).

*Comparisons between treatment groups to vehicle*: There was no difference between vehicle and all other dose group RPLA LA values at any timepoint. The overall ANOVA *P*-value for RPLA LA was 0.8430 (Table 2).

#### Diastolic LV internal diameter in short-axis

There was no difference in LVIDd Sx values between Baseline and any other timepoint across dose groups (Table 1). Therefore, a two-way repeated measures ANOVA test was not performed for this variable.

#### Systolic LV internal diameter in short-axis

**Figure 3:**
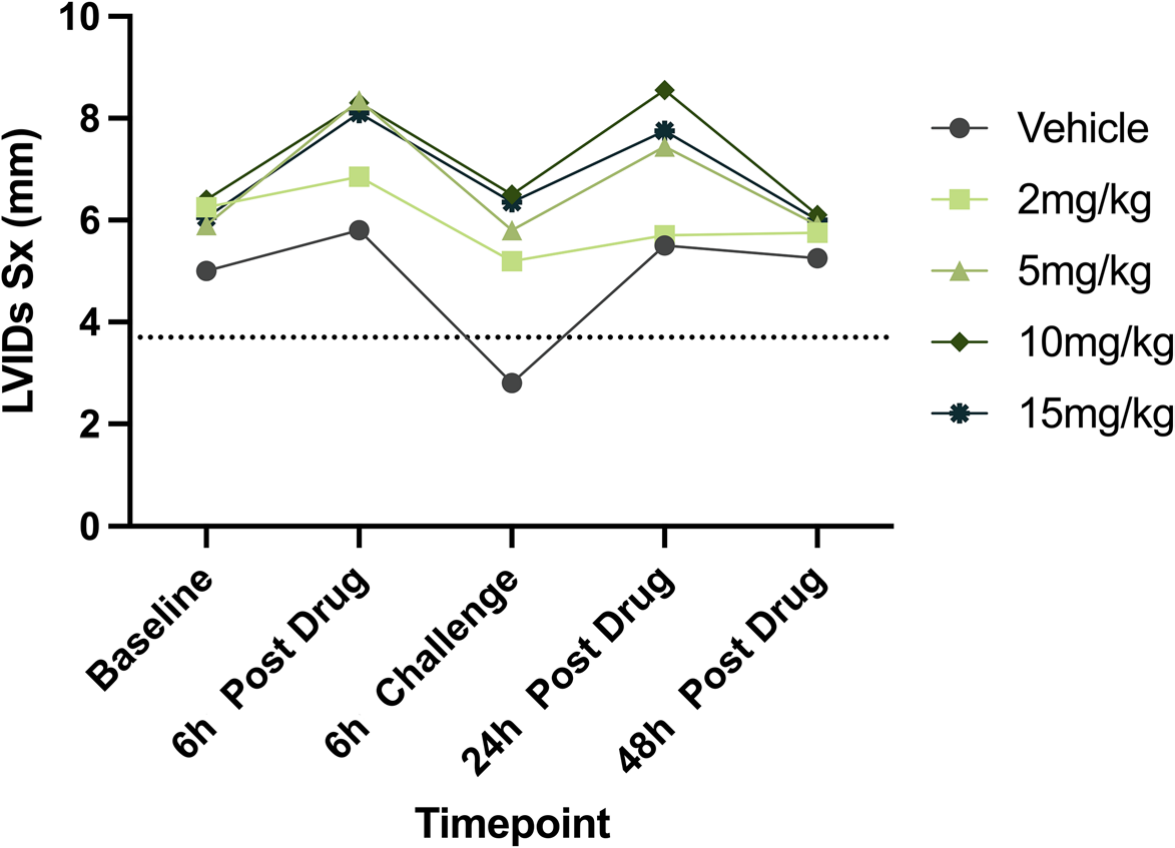
Changes in measures of LV systolic chamber size across timepoints. Median LVIDs Sx values in six cats across Vehicle and four CK-586 oral doses (2-, 5-, 10-, and 15 mg/kg) spanning a 48 h time period are illustrated. Dashed line represents lower-bound cut-off for normal feline LVIDs Sx measurement (3.7 mm)^36^. *LVIDs Sx* systolic left ventricular internal diameter in short-axis, *h* hour.

*Comparisons within treatment groups to baseline*: Drug treatment resulted in significantly increased LVIDs Sx values in the 15 mg/kg dose group (*P*_adjusted_ = 0.0325; Fig. 3 and Table 1). No other dose group had significant differences in LVIDs Sx values between Baseline and any other timepoint.

*Comparisons between treatment groups to vehicle*: At the 6 h Dobutamine Challenge timepoint, values for LVIDs Sx were significantly greater in the 2- and 15 mg/kg dose group when compared to Vehicle (*P* = 0.0369 and *P* = 0.005, respectively). The overall ANOVA *P*-value for LVIDs Sx was 0.0786 (Table 2).

### LV systolic function

#### LV fractional shortening

**Figure 4:**
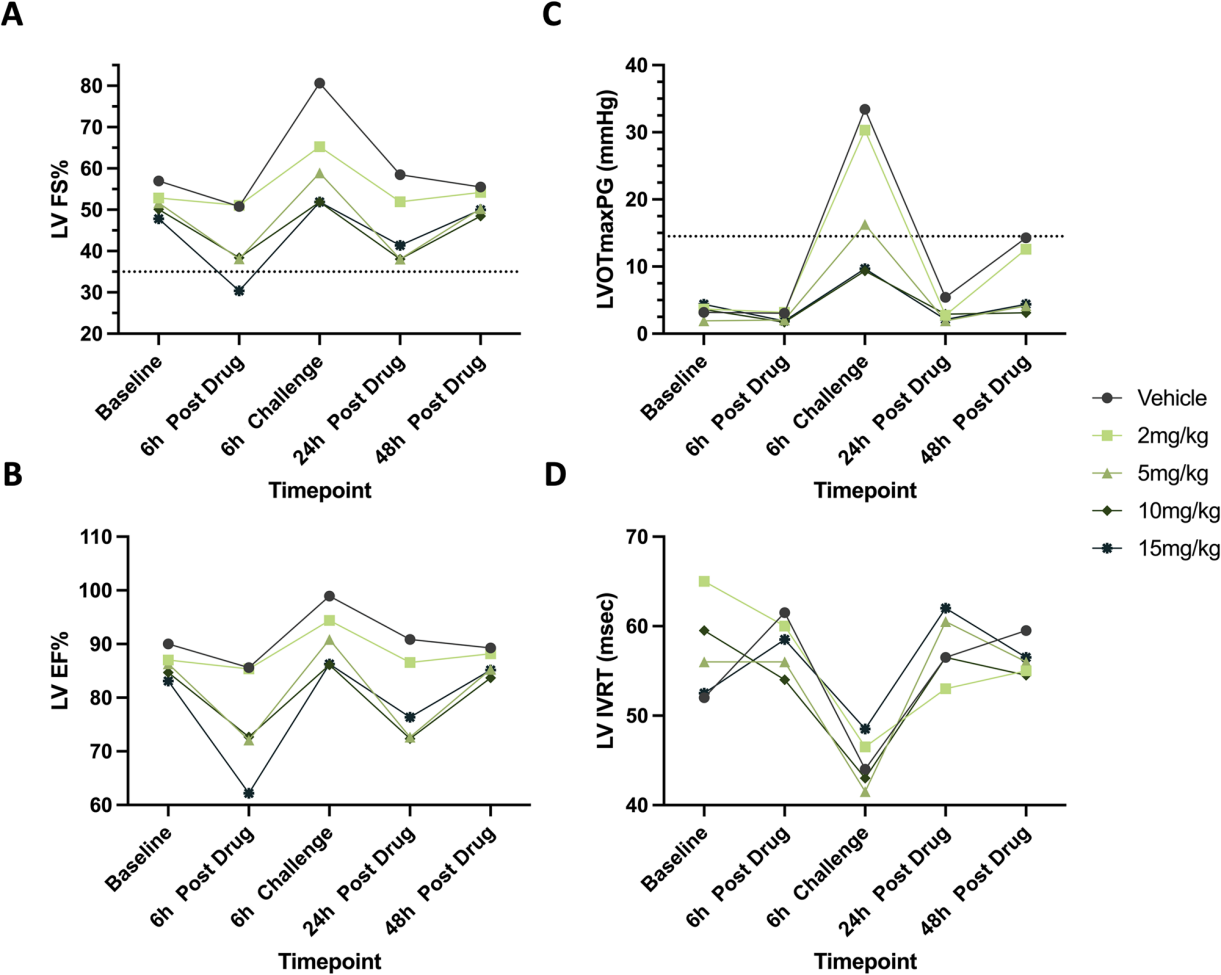
Changes in measures of LV function and LV pressure gradient across timepoints. Median LV FS% (**A**), LV EF% (**B**), LVOTmaxPG (**C**), and LV IVRT (**D**) values in six cats across Vehicle and four CK-586 oral doses (2-, 5-, 10-, and 15 mg/kg) spanning a 48 h time period are illustrated. Dashed line represents the lower-bound cut-off for normal feline LV FS% measurements (35%) and the upper-bound cut-off LVOTmaxPG (14.5 mmHg)^12,14,32^. *LV* left ventricular, *FS%* percent fractional shortening, *EF%* percent ejection fraction, *LVOTmaxPG* left ventricular outflow tract maximum pressure gradient, *IVRT* isovolumetric relaxation time, *h* hour.

*Comparisons within treatment groups to baseline*: In Vehicle-treated cats, dobutamine challenge resulted in significantly increased LV FS% values (*P*_adjusted_ = 0.0423). Administration of 2-, 5-, 10-, and 15 mg/kg CK-586 resulted in resolution of dobutamine-induced (6 h Challenge) increases in LV FS% seen in cats administered Vehicle. Additionally, at 15 mg/kg, LV FS% was significantly reduced at the 6 h Post Drug timepoint (*P*_adjusted_ = 0.0423; Fig. 4A and Table 1).

*Comparisons between treatment groups to vehicle*: Values for LV FS% were significantly higher when cats were given Vehicle as opposed to 15 mg/kg of CK-586 at the 6 h Post Drug and 6 h Challenge timepoints (*P* = 0.0111 and *P* = 0.0004, respectively). Vehicle LV FS% values were also statistically greater at the 24 h Post Drug timepoint than that of the 10 mg/kg dose group (*P* = 0.0282). The overall ANOVA *P*-value for LV FS% was 0.0024 (Table 2).

#### LV ejection fraction

*Comparisons within treatment groups to baseline*: Similar to LV FS%, dobutamine challenge resulted in significantly increased LV EF% values in the Vehicle group (*P*_adjusted_ = 0.0423). Administration of 2-, 5-, 10-, and 15 mg/kg CK-586 resulted in resolution of dobutamine-induced (6 h Challenge) increases in LV EF%. 6 h Post Drug LV EF% values were statistically decreased compared to Baseline values in the 15 mg/kg dose group (*P*_adjusted_ = 0.0423; Fig. 4B and Table 1).

*Comparisons between treatment groups to vehicle*: Vehicle versus 15 mg/kg LV EF% values were statistically significant at the 6 h Post Drug and 6 h Challenge timepoint (*P* = 0.0106 and *P* = 0.0154, respectively), with 15 mg/kg resulting in reduced LV EF%. LV EF% values between the Vehicle and 10 mg/kg group were also statistically reduced in the 10 mg/kg group at the 24 h Post Drug timepoint (*P* = 0.0209). The overall ANOVA *P*-value for LV EF% was 0.0034 (Table 2).

#### LVOT maximum pressure gradient

*Comparisons within treatment groups to baseline*: Dobutamine challenge significantly increased LVOTmaxPG values when compared to Baseline values in the Vehicle-, 2 mg/kg-, and 5 mg/kg-treated cats (*P*_adjusted_ = 0.0021, *P*_adjusted_ = 0.0423, and *P*_adjusted_ = 0.0139, respectively). LVOTmaxPG was blunted when challenged with dobutamine in the setting of 10- and 15 mg/kg of CK-586. Treatment with a single 15 mg/kg dose of CK-586 successfully decreased LVOTmaxPG values 6 h post-treatment (*P*_adjusted_ = 0.0139; Fig. 4C and Table 1).

*Comparisons between treatment groups to vehicle*: No comparisons between Vehicle and 2-, 5-, 10-, and 15 mg/kg LVOTmaxPG values were statistically significant from each other at any timepoint. The overall ANOVA *P*-value for LVOTmaxPG was 0.1307 (Table 2).

#### Mitral valve E/A, LV isovolumetric relaxation times, and left auricular flow

Within-dose differences in MV E/A, LV IVRT, and/or Lau values between Baseline and other study timepoints were not observed via Dunn’s multiple comparisons testing in the Vehicle, 2-, 5-, 10-, and 15 mg/kg groups. However, the LV IVRT variable resulted in an overall statistically significant Friedman *P*-values across dose groups (Table 1 and Fig. 4D). Thus, to assess between-timepoint LV IVRT value differences between Vehicle and treatment doses that would otherwise not be captured via Friedman and Dunn’s testing, a two-way repeated measures ANOVA test was performed for only this variable. No comparisons between Vehicle and 2-, 5-, 10-, and 15 mg/kg LV IVRT values were statistically significant from each other at any timepoint; the overall ANOVA *P*-value for LV IVRT was 0.9206 (Table 2).

#### Incidence of obstruction

**Figure 5:**
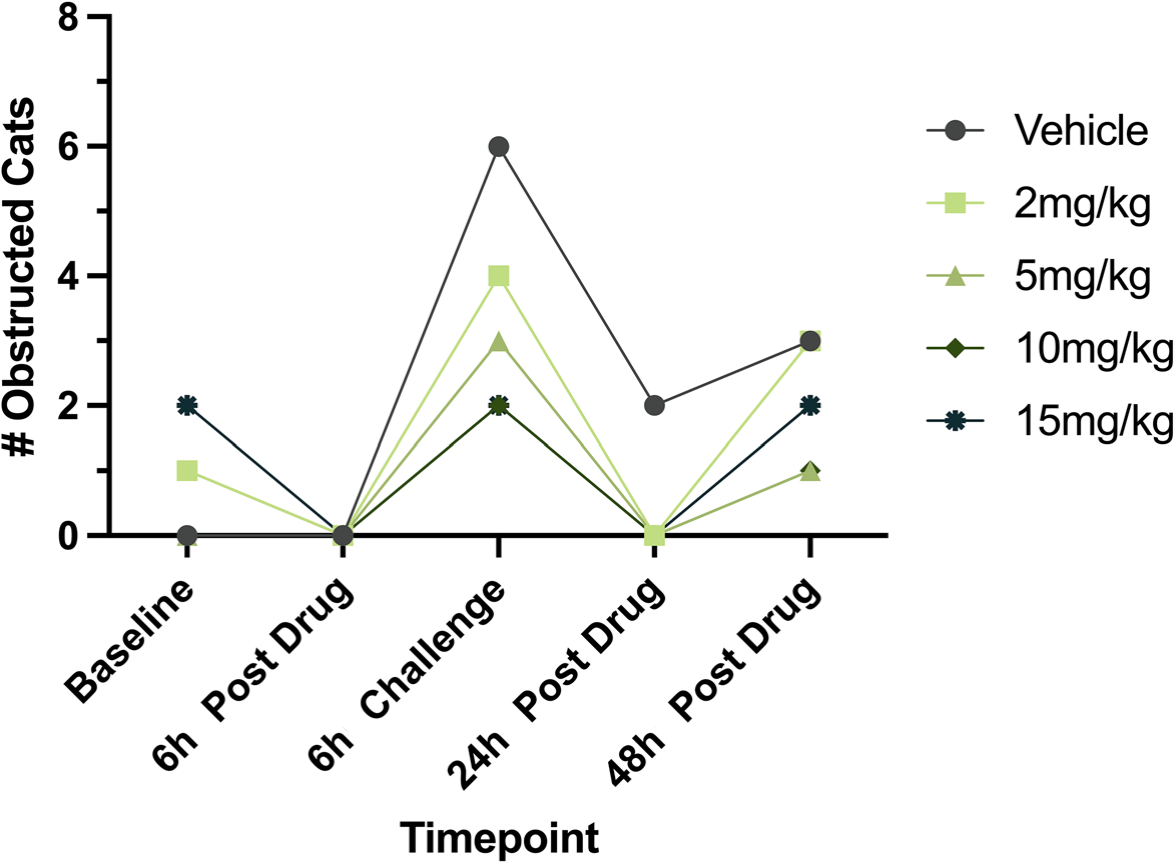
Observed incidence of LVOTO across timepoints. The total number of observed LVOTO incidences defined by an LVOT velocity > 1.9 m/s (LVOTmaxPG ≥ 14.5 mmHg)^12,32^ in six cats across Vehicle and four doses of CK-586 (2-, 5-, 10-, and 15 mg/kg). *LVOTO* left ventricular outflow tract obstruction, *LVOT* left ventricular outflow tract, *LVOTmaxPG* left ventricular outflow tract maximum pressure gradient, *h* hour.

A decrease in the incidence of obstruction with an increase in CK-586 dose was observed at each timepoint (Fig. 5); however, the distribution of the observed incidence of obstruction between different timepoints was not statistically different across any of the doses (Table 3).

**Table 3:** Incidence of observed LVOTO across vehicle and treatment groups per timepoint.

Dose	Timepoint	# Obstructed	# Not Obstructed	*P*-value
Vehicle	Baseline	0	6	–
	6 h Post Drug	0	6	–
	6 h Challenge	6	0	–
	24 h Post Drug	2	4	–
	48 h Post Drug	3	3	–
2 mg/kg	Baseline	1	5	1
	6 h Post Drug	0	6	1
	6 h Challenge	4	2	0.4545
	24 h Post Drug	0	6	0.4545
	48 h Post Drug	3	3	1
5 mg/kg	Baseline	0	6	1
	6 h Post Drug	0	6	1
	6 h Challenge	3	3	0.1818
	24 h Post Drug	0	6	0.4545
	48 h Post Drug	1	5	0.5455
10 mg/kg	Baseline	1	5	1
	6 h Post Drug	0	6	1
	6 h Challenge	2	4	0.0606
	24 h Post Drug	0	6	0.4545
	48 h Post Drug	1	5	0.5455
15 mg/kg	Baseline	2	4	0.4545
	6 h Post Drug	0	6	1
	6 h Challenge	2	4	0.0606
	24 h Post Drug	0	6	0.4545
	48 h Post Drug	2	4	1

Results from a 2 × 2 Fisher’s exact test on the observed incidence of LVOTO in cats receiving Vehicle and CK-586 at four doses (2-, 5-, 10-, and 15 mg/kg) across study timepoints are reported.

*h* hour.

## Discussion

Treatment with oral CK-586, a novel cardiac myosin inhibitor, safely ameliorated LVOTO in oHCM cats. In this study, we report the beneficial effects of CK-586 treatment at eliminating obstruction (reducing LVOTOmaxPG), increasing measures of systolic chamber size (LVIDs Sx), and thus, decreasing select measures of heart function (LV FS% and LV EF%) in the absence of impact on HR. Dose-dependent effects were observed for the aforementioned echocardiographic changes after drug administration in spite of dobutamine challenge. The greatest effects were observed at the 6 h Post Drug timepoint for the LV FS%, LV EF%, and LVOTmaxPG variables. Pharmacodynamic similarities were observed between the 10- and 15 mg/kg doses for LVIDS Sx, LV FS%, and LVOTOmaxPG values. Similar to other cardiac myosin inhibitors, dose dependency of hemodynamic functional responses were observed. When dobutamine was administered at the 6 h timepoint, protection against obstruction was apparent in the 10- and 15 mg/kg dose groups. Median LVOTmaxPG values remained below the obstruction threshold (14.5 mmHg) in the 5-, 10-, and 15 mg/kg dose groups up until the 48 h Post Drug timepoint; in the Vehicle and 2 mg/kg dose group, values approaching this cut-off were noted. The Vehicle and 2 mg/kg group exhibited similar pharmacodynamic characteristics, particularly for median LVOTmaxPG and LV EF% values. Collectively, this data suggests that the > 5 and < 15 mg/kg dose range of CK-586 represents a promising target dose for the treatment of obstruction and feline subjects with oHCM. Of note, when cats received a single 15 mg/kg dose of CK-586, median 6 h Post Drug LV FS% values fell below the lower-bound cut-off point (35%) suggesting this level of dose escalation in cats is not desirable.

Cardiac myosin inhibitors, mavacamten and aficamten, have previously been evaluated in the same cat model^27,28,35^. Because each study was conducted under different experimental conditions, it is difficult to make direct comparisons between these three cardiac myosin inhibitors in this model. For example, mavacamten was administered by IV infusion, echocardiographic assessment was performed in fully anesthetized (alfaxalone and midazolam induction with isoflurane and oxygen maintenance) cats, and isoproterenol was administered to elevate HR and induce previously observed LVOTO to pre-anesthetized values^35^. In the current study, CK-586 was administered orally and LVOTO was induced by dobutamine. Overall, like mavacamten and aficamten, CK-586 demonstrated dose- and exposure-related reductions in measures of systolic contractility and LVOTO and further provides support for cardiac myosin inhibitors as a potential therapeutic approach for cats with oHCM.

The present study only included cats in the B1 stage of their disease; therefore, the results from this study are only applicable to cats that have yet to progress into advanced subclinical (i.e., stage B2 [subclinical LV hypertrophy with evidence of left atrial enlargement]) and/or symptomatic disease states (i.e., left-sided CHF). Whether CK-586 has the propensity to mitigate obstruction at different disease stages, particularly in patients that have progressed onto CHF, remains unknown and warrants further investigation. However, results from human cardiac myosin inhibitors trials suggest that symptom treatment is feasible. While echocardiographic assessment of systolic function remained normal throughout this study, invasive hemodynamic assessments and measurements of blood pressure were not performed.

This is a hypothesis-driven, non-GLP, exploratory study assessing the pharmacodynamic effects of CK-586 in six cats serving as their own controls across five different study groups. Although this design helps to limit the challenges of a smaller sample size, the possibility of type-II errors cannot be excluded; this may be evidenced by the trends in study variables that did not meet statistical significance. The effect sizes in this repeated-measures study appear biologically meaningful, however, this study did not specifically evaluate intra-day or inter-day precision or accuracy. This study highlights the promising short-term effects of CK-586 dosing in asymptomatic oHCM cats. As such, long-term and disease outcomes data cannot be extrapolated from the results presented here. Further studies investigating the extent to which CK-586 administration may benefit cats with oHCM is needed. Lastly, the cats in this study exhibited oHCM at rest but were sedated for the evaluations of this study, necessitating the use of a dobutamine challenge to provoke reliable obstruction. Ideally, future chronic dosing studies would assess drug impact in the resting and provoked state without the variable of sedation.

### Supplementary Information

Supplementary Information.

## Data Availability

Data is available upon reasonable request from the corresponding author.

## Abbreviations

ANOVA: Analysis of covariance
API: Active pharmaceutical ingredient
AUC: Area under the curve
ASH: Asymmetric septal hypertrophy
CHF: Congestive heart failure
Cmax: Maximum concentration
CmaxD: Dose-normalized maximum concentration
ECG: Electrocardiogram
EF%: Percent ejection fraction
FS%: Percent fractional shortening
HCM: Hypertrophic cardiomyopathy
HR: Heart rate
h: Hour
IACUC: Institutional Animal Care and Use Committee
IV: Intravenous
IVRT: Isovolumetric relaxation time
Kel: Elimination rate constant
LA: Left atrium/atrial
Lau: Left auricular flow velocity
LC-MS/MS: Liquid chromatography-tandem mass spectrometry
LV: Left ventricle/ventricular
LVIDd Sx: Short-axis diastolic left ventricular internal diameter
LVIDs Sx: Short-axis systolic left ventricular internal diameter
LVOT: Left ventricular outflow tract
LVOTmaxPG: Left ventricular outflow tract maximum pressure gradient
LVOTO: Left ventricular outflow tract obstruction
MYBPC3: Myosin-binding protein C3
MV E/A: Mitral valve passive filling/active filling ratio
oHCM: Obstructive hypertrophic cardiomyopathy
RPLA: Right parasternal long-axis
RPLA LA: Maximal right parasternal long-axis left atrial diameter
Tmax: Time-to-maximum plasma concentration
