# Supplementary material for: Cardiac myosin inhibitor, CK-586, minimally reduces systolic function and ameliorates obstruction in feline hypertrophic cardiomyopathy
Source: Sci Rep. 2024 May 27;14:12038. doi: 10.1038/s41598-024-62840-3 (PMC11130313; doi:10.1038/s41598-024-62840-3)
Supplement: Supplementary file 1 — Supplementary Information. [file 41598_2024_62840_MOESM1_ESM.docx]

| Subject | Sex | Age (yr) | A31P Genotype | Body Weight (kg) | Dose (mg/kg) |
| --- | --- | --- | --- | --- | --- |
| **Pharmacokinetics/Dose-Finding** | | | | | |
| Cat 1 | M | 3.08 | Heterozygous | 5.4 | 10 |
| Cat 2 | F | 1.17 | Wildtype | 3.2 | 10 |
| Cat 3 | M | 1.17 | Heterozygous | 4.7 | 10 |
| Cat 4 | F | 3.17 | Wildtype | 5.8 | 10 |
| Cat 5 | F | 3.25 | Heterozygous | 4.8 | 3 |
| Cat 6 | F | 1.42 | Heterozygous | 2.8 | 3 |
| Cat 7 | M | 3.08 | Heterozygous | 4 | 3 |
| Cat 8 | M | 1.75 | Heterozygous | 4.8 | 3 |
| Cat 9 | F | 3.52 | Heterozygous | 3.6 | 16.2 |
| Cat 10 | F | 1.21 | Wildtype | 3.4 | 15.9 |
| Cat 11 | M | 1.76 | Wildtype | 4.5 | 10.5 |
| Cat 12 | M | 1.21 | Heterozygous | 6.7 | 4.8 |
| **Pharmacodynamics** | | | | | |
| Cat 13 | M | 6.17 | Heterozygous | 4.93 | 2, 5, 10, 15 |
| Cat 14 | M | 5.5 | Heterozygous | 7.49 | 2, 5, 10, 15 |
| Cat 15 | M | 3.67 | Homozygous | 6.45 | 2, 5, 10, 15 |
| Cat 16 | M | 1.83 | Heterozygous | 4.8 | 2, 5, 10, 15 |
| Cat 17 | M | 5.5 | Heterozygous | 5.4 | 2, 5, 10, 15 |
| Cat 18 | F | 2.25 | Heterozygous | 4.39 | 2, 5, 10, 15 |

**Supplemental Table 1. Demographic data of study cats.** The sex, age, A31P status, body weight, and dose specifications for all cat pharmacokinetic/dose-finding and pharmacodynamic analyses are presented.

| Dose  mg/kg (umol/kg) | Subject | T_1/2_  (min) | AUClast  (min*umol/L) | AUClast/Dose  (min*kg*umol/L/umol) | Cmax  (umol/L) | Tmax  (min) |
| --- | --- | --- | --- | --- | --- | --- |
| 16.2 (39.96) | Cat 9 | ND | 1630 | 40.8 | 1.52 | 480 |
| 15.9 (39.22) | Cat 10 | ND | 953 | 24.3 | 1.37 | 240 |
| 10.5 (25.9) | Cat 11 | ND | 838 | 32.4 | 0.89 | 480 |
| 4.8 (11.8) | Cat 12 | ND | 461 | 39.1 | 0.43 | 480 |
| 10 (24.7) | Cat 1 | ND | 1534 | 62.1 | 1.88 | 240 |
|  | Cat 2 | ND | 935 | 37.9 | 1.3 | 240 |
|  | Cat 3 | ND | 881 | 35.7 | 0.83 | 480 |
|  | Cat 4 | ND | 804 | 32.6 | 0.82 | 1440 |
|  | **N** | ND | 4 | 4 | 4 | 4 |
|  | **Mean** | ND | 1039 | 42 | 1.21 | 600.00 |
|  | **SD** | ND | 335 | 14 | 0.50 | 571.31 |
|  | **CV%** | ND | 32 | 32 | 42 | 95 |
| 3 (7.4) | Cat 5 | ND | 289 | 39.1 | 0.3 | 240 |
|  | Cat 6 | 1016 | 227 | 30.7 | 0.24 | 90 |
|  | Cat 7 | ND | 288 | 38.9 | 0.35 | 240 |
|  | Cat 8 | 1016 | 227 | 30.7 | 0.24 | 90 |
|  | **N** | 2 | 4 | 4 | 4 | 4 |
|  | **Mean** | 1016 | 258 | 35 | 0.28 | 165.00 |
|  | **SD** | 0 | 36 | 5 | 0.05 | 86.60 |
|  | **CV%** | 0 | 14 | 14 | 19 | 52 |

**Supplemental Table 2. Noncompartmental Analysis Summary.** Pharmacokinetics parameters are presented. ND= not determined (no terminal phase was seen).

**Abbreviations:** T_1/2_= half-life; AUClast= area under curve last; AUClast/Dose= dose-normalized area under curve last; Cmax= maximum concentration; Tmax= time-to-maximum; ND= not determined; SD= standard deviation; CV%= coefficient of variation percentage.

**Supplemental Figure 1. CK-586 decreased rat left ventricular fractional shortening in a concentration-related manner.** Coupled with the pharmacokinetic data in cats, the pharmacokinetic-pharmacodynamic relationship in rats was used to determine doses for the pharmacodynamic assessment in the A31P cat colony. Data is shown as normalized, mean fractional shortening values (±SD) as a function of total CK-586 plasma concentration.
